# Caring for Caregivers: System-Level Solutions to Moral Injury in Burn Teams

**DOI:** 10.1093/jbcr/irag069

**Published:** 2026-05-07

**Authors:** Joshua Khorsandi, Jason Mirharooni, Joshua Ahdout, Justin Kahen, Eli Nazarian, Demitri Franzoni, Joshua MacDavid

**Affiliations:** Department of Plastic and Reconstructive Surgery, Kirk Kerkorian School of Medicine at University of Nevada Las Vegas, Las Vegas, NV 89106, United States; Department of Surgery, Florida International University Herbert Wertheim College of Medicine, Miami, FL 33199, United States; Department of Plastic and Reconstructive Surgery, Kirk Kerkorian School of Medicine at University of Nevada Las Vegas, Las Vegas, NV 89106, United States; Department of Plastic and Reconstructive Surgery, Kirk Kerkorian School of Medicine at University of Nevada Las Vegas, Las Vegas, NV 89106, United States; Department of Plastic and Reconstructive Surgery, Kirk Kerkorian School of Medicine at University of Nevada Las Vegas, Las Vegas, NV 89106, United States; Department of Plastic and Reconstructive Surgery, University of Nevada Las Vegas, Las Vegas, NV 89106, United States; Department of Plastic and Reconstructive Surgery, University of Nevada Las Vegas, Las Vegas, NV 89106, United States

**Keywords:** moral distress, moral injury, compassion fatigue, burn care teams, organizational ethics, clinician well-being

## Abstract

Burn care teams—nurses, physicians, rehabilitation therapists, respiratory therapists, and unlicensed care providers such as burn technicians or patient care assistants—work amid catastrophic injury, disfigurement, severe pain, and complex end-of-life decisions, creating high risk for moral distress, moral injury, and compassion fatigue. This structured, PRISMA-informed narrative review synthesized burn-specific evidence and literature from analogous high-acuity settings. PubMed, CINAHL, PsycINFO, and Google Scholar were searched (English, 2010-2025; foundational earlier works retained). Eligible publications addressed ethically mediated workforce outcomes and organizational, systemic, or team-level drivers and responses; resilience-only studies were excluded unless conceptually informative. Burn-focused studies were sparse but aligned with ICU findings: antecedents included resource scarcity and staffing mismatch, perceived futility and contested goals of care, hierarchical role conflict and constrained voice, and ethically unsafe climates. Consequences clustered around emotional exhaustion, secondary traumatic stress, moral residue, turnover intention, and downstream risks to safety and quality. Across settings, the most promising protections were organizational: accessible leadership, safe staffing and predictable workload, psychologically safe communication, structured peer support and debriefings, reflective forums (eg, Schwartz Rounds), and proactive ethics consultation and moral distress consultation services that surface remediable policy and culture gaps. Moral suffering in burn units should be treated as an organizational ethics problem; embedding moral protection into governance, quality metrics, and ethics infrastructure is essential. Such system-level approaches can reduce preventable moral harm, sustain workforce retention, and protect patient and family experience in burn centers.

## INTRODUCTION

Burn care is delivered by a multidisciplinary team that includes nurses, physicians (including residents and attending physicians), rehabilitation and respiratory therapists, and unlicensed care providers such as burn technicians or patient care assistants (PCAs), all of whom work in close proximity to severe injury, disfigurement, and complex end-of-life decision-making. Unlicensed care providers, including burn technicians or PCAs, play a central role in daily wound care and are frequently exposed to high levels of patient suffering, yet remain underrepresented in discussions of team-based moral distress and often lack formal organizational support structures. Attending physicians may also experience distinct moral strain related to their ultimate responsibility for clinical outcomes.

Care unfolds amid unstable physiology, painful procedures, and prolonged trajectories of recovery. Burn-center evidence suggests that clinicians, especially nurses, report high moral distress, emotional exhaustion, and intent to leave, driven by repeated exposure to suffering and constrained decision-making authority.^1^ Burn ethics analyses similarly highlight moral tension around triage, resource allocation, and consent.^2^ Burn teams face repeated decisions about grafting, rehabilitation priorities, and goals of care that compound moral residue.

Across critical care and oncology, moral distress is commonly defined as psychological anguish that arises when clinicians know the ethically appropriate action but are constrained from acting.^3^^,^^4^ More recent scholarship distinguishes moral distress from moral injury: while moral distress arises from constraints that prevent acting on one’s ethical beliefs, moral injury refers to a deeper and often enduring harm to one’s moral identity following perceived participation in morally troubling events.^5^ Compassion fatigue describes the erosion of empathic capacity with chronic exposure to others’ trauma.^6^ These overlapping constructs are linked to burnout, depression, and posttraumatic symptoms, with implications for patient safety.^7^^,^^8^

Reviews demonstrate that moral distress is especially prevalent in intensive care units, where life-sustaining treatments, end-of-life conflicts, and resource constraints are concentrated.^9^ Yet only a small subset of this literature focuses on burn teams, and even fewer studies frame the prevention of moral injury and compassion fatigue as an organizational ethics obligation. Interventions are often framed as individual resilience or self-care, with less attention to workload, staffing, ethical climate, and governance.^10^^,^^11^ Emerging work on moral distress consultation, institutional ethics resources, and moral resilience supports shifting from individualizing moral suffering to recognizing it as a signal of systems-level value conflict.^12^

Accordingly, this structured, PRISMA-informed narrative review synthesizes evidence on antecedents, consequences, and system-level responses to moral distress, moral injury, and compassion fatigue among burn care staff and clinicians in analogous high-acuity environments. We argue these phenomena are organizational ethics problems, not only issues of individual coping. We highlight strategies—leadership behaviors, ethical climate, peer support and debriefing, and ethics consultation—that can mitigate moral injury risk, and we identify gaps in conceptualizing moral protection as an institutional duty in burn care.

## METHODS

A structured, narrative review was conducted using PRISMA-informed principles to identify empirical and conceptual literature on moral distress, moral injury, and compassion fatigue among burn care clinicians and staff in analogous high-acuity settings. Searches were performed in PubMed (https://pubmed.ncbi.nlm.nih.gov; accessed on September 25, 2025), CINAHL, PsycINFO, and Google Scholar (https://scholar.google.com; accessed on September 25, 2025). Search terms included combinations of “moral distress,” “moral injury,” “compassion fatigue,” “burn care,” “burn unit,” “burn ICU,” “critical care ethics,” “clinician well-being,” “ethical climate,” “organizational ethics,” “nursing ethics,” and “secondary traumatic stress.” Boolean operators and controlled vocabulary were used when applicable to optimize retrieval.

Search results were restricted to English-language publications from 2010 to 2025 in order to capture contemporary developments in the conceptualization of moral injury and emerging organizational ethics frameworks relevant to healthcare systems. Earlier seminal works were retained when foundational to understanding the evolution of these constructs or when they continued to influence current practice and scholarship. Reference lists of included studies and major reviews were examined to identify additional relevant sources.

Eligibility for inclusion was based on the relevance of each publication to ethically mediated workforce outcomes, its applicability to burn care or comparable intensive care contexts, and its attention to the organizational, systemic, or team-level conditions that shape moral distress and moral injury. Studies that focused exclusively on individual coping or resilience training without situating these within broader structural factors were excluded unless they provided conceptual contrasts useful for interpreting system-level interventions. Empirical studies, conceptual analyses, ethics case reports, integrative and systematic reviews, and evaluations of organizational programs such as debriefings, peer support, or ethics consultation were all eligible for inclusion. In total, 48 peer-reviewed studies met inclusion criteria and were integrated into the final narrative synthesis.

The final body of literature comprised a diverse set of burn-specific empirical studies, high-acuity nursing research, and organizational ethics scholarship. Data were extracted and synthesized narratively, with particular attention to identifying recurring antecedents of moral distress and moral injury, the consequences for clinician well-being and patient care, and the organizational strategies reported to mitigate or exacerbate these phenomena. This approach allowed for the integration of evidence across heterogeneous study designs while maintaining a consistent focus on system-level contributors to moral suffering in burn care.

## RESULTS

### Overview of included literature and relevance to burn care

The literature directly focused on moral distress and compassion fatigue in burn units was relatively sparse but highly consistent with broader findings from adult and pediatric intensive care settings. A pilot study from a US burn center reported moderate to high moral distress among nurses, with frequent exposure to futile or nonbeneficial treatments, inadequate staffing, and conflicts with physicians cited as key triggers.^1^ A narrative review of quantitative evidence on compassion fatigue in burn units synthesized data from several small studies and found substantial rates of secondary traumatic stress and burnout among both nurses and physicians, closely linked to cumulative exposure to severe injuries, pain, disfigurement, and grief.^13^ A recent quality-improvement report from a burn/trauma ICU described decreased self-reported moral distress after implementation of interprofessional “ethics huddles,” suggesting that structured, unit-level ethical dialogue may be feasible and acceptable in burn environments.^14^

### Antecedents: resource scarcity, clinical complexity, and ethical climate

Across studies, recurrent antecedents of moral distress, moral injury, and compassion fatigue clustered around resource constraints, clinical futility, role conflict, and organizational ethical climate. Intensive care unit nurses consistently identified inadequate staffing, bed shortages, and lack of appropriate resources or rehabilitation options as precipitants of moral distress, particularly when they believed that patient care fell below professional standards or that preventable suffering was occurring.^15^^,^^16^ For burn teams, such constraints are amplified by the intensive resource demands of large total body surface area injuries, long ICU stays, and the high costs of reconstructive procedures. Qualitative accounts from burn nurses describe distress when rationing operating-room time or blood products, or when delayed transfers from referring hospitals result in worse outcomes than might have been achievable with earlier intervention.^1^^,^^2^

Perceived futility and conflicts around life-sustaining treatments emerged as another central antecedent. Critical care nurses report high distress when continuing aggressive interventions in patients with very low likelihood of meaningful recovery, especially when they perceive family requests or institutional policies, not clinical judgment, to be driving decisions.^9^^,^^17^ Burn specialists encounter analogous scenarios when repeatedly returning patients to the operating room for major grafting procedures despite multiorgan failure, or when facial and hand burns raise questions about the long-term quality of life in the context of poor neurologic prognosis. Such situations can be experienced as potential morally injurious events when clinicians feel compelled to participate in care they regard as inhumane, nonbeneficial, or contrary to their core professional commitments.^5^^,^^18^

Repeated exposure to intense suffering, disfigurement, and traumatic death contributes to compassion fatigue and moral injury risk. Higher exposure to trauma, resuscitations, and end-of-life events is associated with secondary traumatic stress and burnout in critical care clinicians.^19^ In burn units, the sensory intensity of care, including disfigurement, pain behaviors, and complex wound management, may further compound these effects; burn-specific evidence links frequent exposure to severe injury and death with emotional exhaustion and reduced compassion satisfaction.^13^

Role conflict, limited authority, and unclear responsibility lines are recurrent organizational antecedents of moral distress. Moral distress increases when clinicians are required to implement care they judge unethical or when hierarchical cultures constrain meaningful deliberation and speaking up.^10^^,^^20^ Burn teams may be particularly vulnerable when goals of care are contested across services or when clinicians are held responsible for advocacy without commensurate decision-making authority.^1^^,^^12^

Ethical climate emerged as a cross-cutting modifier of moral distress. Supportive climates characterized by open communication, mutual respect, and responsiveness to staff concerns are associated with lower moral distress and burnout, whereas blame-oriented or silencing cultures exacerbate distress.^21^ In burn units, treating ethics as compliance rather than shared practice may intensify moral distress by limiting opportunities for reflection and dialogue. Table 1 summarizes the principal ethical domains contributing to moral distress and moral injury in burn care teams, outlining key burn-care contexts, their ethical significance, and representative sources from the literature.

**Table 1:** Ethical Domains Contributing to Moral Distress and Moral Injury in Burn Care Teams

Domain	Burn-care context	Ethical significance	Representative source
Resource scarcity and workload	High-acuity burns require prolonged ICU stays, repeated surgeries, and intensive staffing	Resource constraints heighten moral distress when clinicians perceive preventable suffering or compromised standards of care	Leggett et al.^1^
Perceived futility and goals-of-care conflict	Continued grafting or life-sustaining treatment despite poor prognosis	Participation in perceived nonbeneficial care contributes to moral distress, moral residue, and moral injury	Litz et al.^5^
Exposure to trauma and suffering	Frequent exposure to severe injury, disfigurement, pain, and death	Cumulative trauma increases risk of compassion fatigue and emotional exhaustion	Bayuo & Agbenorku^13^
Role conflict and constrained voice	Hierarchical burn teams with limited nurse or therapist authority	Moral distress increases when clinicians lack power to influence ethically salient decisions	Musto et al.^10^
**Ethical climate**	Ethics treated as compliance rather than shared deliberation	Supportive ethical climates mitigate moral distress; blame-oriented cultures exacerbate it	Hamric et al.^21^

Abbreviation: ICU, intensive care unit.

### Consequences: emotional exhaustion, moral injury, turnover, and safety

Multiple reviews link clinician burnout and poor well-being to adverse patient safety and quality outcomes. Panagioti et al.’s meta-analysis of physician burnout found that higher burnout was associated with increased patient safety incidents, poorer professionalism, and lower patient satisfaction.^7^ Hall et al.’s systematic review similarly reported that poor staff well-being and burnout were associated with more frequent errors and lower safety climate scores across diverse healthcare settings.^8^ A multicenter study of ICU personnel found that average unit moral distress scores were not consistently correlated with measured medication error events, but did highlight substantial variability across disciplines and units, suggesting that some kinds of harm may go unmeasured in routine safety metrics.^22^ Qualitative reports from burn and critical care units describe near-misses, communication breakdowns, and compromised situational awareness when teams are emotionally depleted or disengaged, implying that moral distress may erode safety through complex pathways not fully captured in existing indices.

At the team level, moral distress and compassion fatigue have been associated with reduced cohesion, increased conflict, and diminished trust in leadership. Evaluations of Schwartz Center Rounds—a structured forum for staff to discuss the emotional and ethical challenges of clinical work—report that participants perceive improved understanding of colleagues, enhanced empathy, and greater sense of organizational support, suggesting that prior to such interventions, staff experienced isolation and fragmentation.^23^ Burn-unit staff surveyed in Leggett’s pilot study described feelings of powerlessness, frustration, and moral residue that strained relationships with colleagues and contributed to a sense of working in an ethically unsafe environment.^1^

### Organizational measures and ethics consultation as protective strategies

Across the literature, multilevel organizational measures emerged as the most promising protective strategies against moral distress, moral injury, and compassion fatigue. Supportive leadership—operationalized as leaders who are accessible, transparent, and responsive to staff concerns—was repeatedly associated with lower moral distress and burnout and with better perceptions of ethical climate. Organizational interventions that aligned staffing with patient acuity and created more predictable workloads were also highlighted as foundational; several reviews emphasize that without safe staffing, more targeted interventions have limited impact.^8^^,^^24^

Structured peer support and debriefings constitute a second major category of organizational response. Pilot projects implementing standardized distress debriefings after critical incidents reported high staff satisfaction and perceived reductions in stress, with qualitative feedback emphasizing the value of shared reflection, normalization of emotional responses, and opportunities to identify system improvements.^25^ A narrative review of debriefing and reflective interventions concluded that such programs can mitigate moral distress and improve team communication, particularly when designed to address both ethical and emotional dimensions of cases rather than focusing solely on technical performance.^26^ The RECONN (Reflection and Connection) program, a unit-based intervention that builds social support and moral healing on nursing units, demonstrated improved perceptions of support and reduced moral injury symptoms among participants, highlighting the value of structured, recurring spaces for ethical dialogue and peer connection.

Schwartz Center Rounds are a well-studied model of psychologically safe, multidisciplinary forums for addressing the emotional and ethical challenges of care. Reviews and longitudinal evaluations associate Rounds with reduced distress, increased empathy, improved teamwork, and sustained perceptions of organizational support, signaling institutional recognition of the emotional impact of clinical work.^23^ Although not burn-specific, these forums are readily transferable to burn centers and have been implemented in trauma and critical care settings with comparable moral burdens.

**Figure 1:**
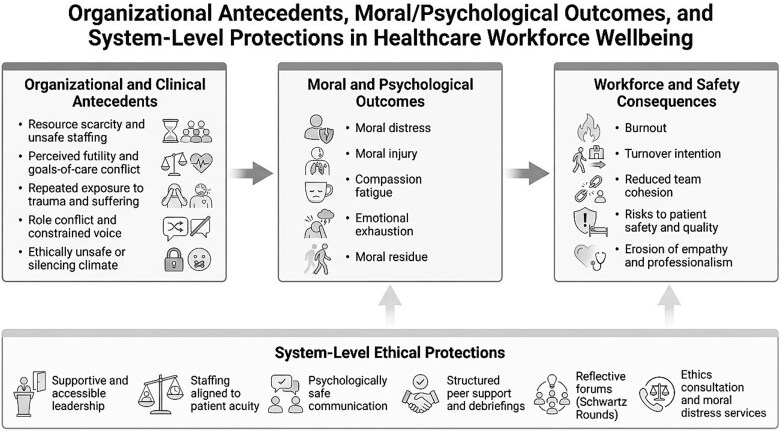
Organizational Framework Linking Clinical and Organizational Antecedents to Moral Distress, Moral Injury, and Compassion Fatigue in Burn Care Teams, With Associated Workforce and Patient Safety Consequences and System-Level Ethical Protections. Image created by authors using FigureLabs.

Ethics consultation and moral distress consultation services represent the most explicit organizational ethics interventions identified. Evaluations of system-level Moral Distress Consultation Services show that consultations frequently surface chronic contributors such as understaffing, communication failures, and policy gaps, and are associated with increased ethical clarity and perceived leadership accountability.^20^ These services reframe moral distress as a signal of system misalignment rather than an individual coping failure. Figure 1 synthesizes organizational antecedents, moral and psychological outcomes, and system-level ethical protections identified across the reviewed literature.

Broader conceptual work on institutional ethics resources argues that health organizations have a duty to create “moral spaces” in which staff, patients, and families can raise ethical concerns, receive support, and participate in deliberation.^12^ Such spaces include ethics committees, consultation services, and educational initiatives that are visible, accessible, and responsive. However, even within the ethics literature, relatively few empiric studies examined proactive ethics consultation in burn or critical care environments; that is, consultation triggered not only by acute conflicts but also by patterns of recurrent moral distress or structural constraints.

### Limitations of resilience training alone

Across systematic reviews of interventions for compassion fatigue, burnout, and moral distress, resilience-oriented programs such as mindfulness training, cognitive-behavioral workshops, and self-care curricula showed modest, short-term improvements in self-reported stress and burnout, but limited or inconsistent effects on moral distress scores or structural drivers of suffering.^27^^,^^28^ A systematic review of mindfulness-based interventions for nurses confirmed small-to-moderate reductions in burnout and compassion fatigue but emphasized that high workload, inadequate staffing, and moral conflict remained largely unaddressed, limiting long-term sustainability.

Conceptual and qualitative work on moral injury further warns that “resilience narratives” can inadvertently individualize what are fundamentally systemic problems, shifting responsibility for adaptation onto clinicians and obscuring organizational accountability.^11^^,^^29^ Articles on moral injury in healthcare emphasize that while personal coping skills and moral resilience are valuable, they cannot substitute for reforms in staffing, workflow, documentation burdens, and governance structures that generate persistent value conflicts.^5^^,^^30^ Our synthesis therefore underscores that resilience training, without accompanying organizational change, risks being experienced by burn clinicians as another demand on limited time rather than a meaningful solution.

### Gaps in conceptualizing organizational ethical obligation

Despite growing recognition of moral injury and moral distress as threats to workforce sustainability, relatively few empirical studies explicitly frame their prevention as an institutional ethical obligation. Nursing ethics scholars argue that organizations have duties not only to avoid harm to patients but also to create conditions for ethical practice and to support nurses’ moral agency.^10^^,^^31^ Yet much of the quantitative literature treats moral distress as an individual-level variable, with organizational factors appearing primarily as correlates rather than as targets for structural intervention. Even moral distress interventions in ICUs often focus on education, communication skills, or staff-level debriefings rather than on policy changes, staffing models, or resource allocation practices.^32^

Within burn care specifically, ethics consultation is rarely evaluated as a proactive resource. The limited burn-unit moral distress interventions described, such as ethics huddles, are promising but small-scale and not formally integrated into institutional ethics infrastructures.^14^ Special issues on ethics in burn care emphasize principles of justice, beneficence, and respect for persons, but seldom analyze organizational responsibility for the moral well-being of the burn workforce.^2^ This gap suggests an opportunity for burn centers and professional societies to articulate explicit ethical standards regarding staff moral safety and to treat moral injury prevention as integral to quality and safety, not merely as a wellness initiative.

## DISCUSSION

**Figure 2:**
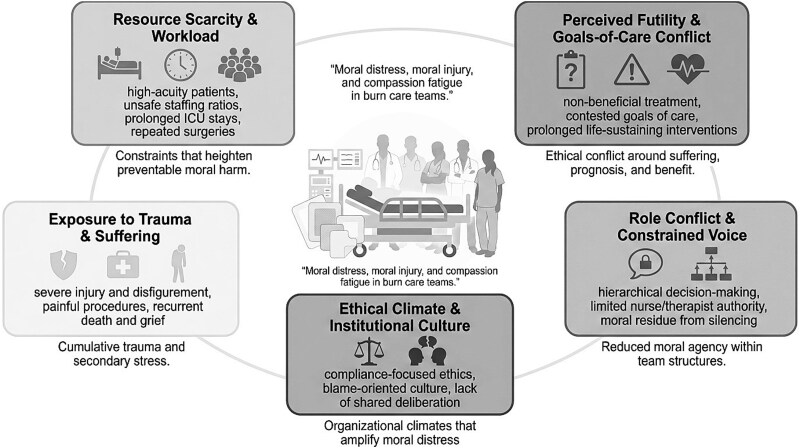
Key Organizational and Clinical Drivers Contributing to Moral Distress, Moral Injury, and Compassion Fatigue in Burn Care Teams, Including Resource Scarcity, Ethical Conflict, Trauma Exposure, Constrained Voice, and Institutional Ethical Climate. Image created by authors using FigureLabs.

This review synthesizes evidence from burn units and analogous high-acuity settings to demonstrate that moral distress, moral injury, and compassion fatigue among burn teams are tightly coupled to organizational conditions. While individual vulnerabilities and coping resources matter, the most consistent antecedents of moral suffering arise from systemic factors: resource scarcity, triage and futility dilemmas, role conflict, and ethical climates that constrain voice. These findings align with conceptual accounts that reframe clinician distress as moral injury rather than burnout alone, highlighting the role of structural value conflicts and institutional betrayals.^5^^,^^11^  Figure 2 synthesizes the primary organizational and clinical drivers of moral distress, moral injury, and compassion fatigue identified across burn care and high-acuity literature.

For the “hidden workforce” of burn nurses, physicians, rehabilitation therapists, and unlicensed care providers, these systemic drivers manifest in distinctive ways. Burn teams routinely navigate ethically fraught decisions about how aggressively to pursue reconstructive surgery in the setting of a poor prognosis, how to prioritize scarce operating-room time, and how to balance painful interventions against uncertain functional gains. They do so in the context of interprofessional hierarchies and complex team structures that can either support or undermine shared moral deliberation. When institutional priorities emphasize throughput, revenue, or reputation over transparent dialogue about limits and trade-offs, clinicians may experience their own moral commitments as misaligned with organizational goals, a core feature of moral injury.^11^^,^^18^

Our synthesis of consequences underscores that moral distress and compassion fatigue are not only workforce well-being issues but also patient safety and quality concerns. The associations between burnout and adverse events, poor professionalism, and lower patient satisfaction are now well established.^7^^,^^8^ In burn units, where staffing is often lean and expertise concentrated, the departure of experienced nurses or therapists due to unresolved moral distress can have outsized effects on quality, disrupting continuity of care and eroding team expertise. Moreover, the psychological toll of moral injury, including guilt, shame, and withdrawal, may compromise clinicians’ capacity for empathic engagement, communication, and situational awareness, with implications for both clinical outcomes and patient/family experience.

The review identifies several organizational strategies associated with reduced moral distress, including supportive leadership, adequate staffing, psychologically safe communication, structured peer support, and timely ethics consultation. Reflective forums such as Schwartz Center Rounds normalize discussion of ethical and emotional challenges and are associated with improved cohesion and perceived organizational support.^23^  Table 2 summarizes system-level and team-based strategies shown to mitigate moral distress and burnout in high-acuity care settings, detailing clinical or organizational contexts, intervention types, key outcomes, and representative sources relevant to burn care. Table 2 summarizes system-level and team-based strategies to mitigate moral distress in burn care teams, outlining clinical and organizational settings, intervention types, key outcomes, and representative sources from the literature.

**Table 2:** System-Level and Team-Based Strategies to Mitigate Moral Distress in Burn Care Teams

Strategy domain	Clinical/organizational setting	Intervention type	Key outcome	Representative source
Supportive leadership and staffing	Burn units and high-acuity ICUs	Leadership accessibility; staffing aligned to acuity	Lower burnout and improved safety and professionalism	Panagioti et al.^7^
Structured peer support and debriefings	ICU and trauma-adjacent settings	Facilitated peer support and postevent debriefings	Reduced emotional distress and improved team functioning	Mealer et al.^33^
Reflective forums (Schwartz Rounds)	Multidisciplinary hospital settings	Regular facilitated reflection sessions	Reduced psychological distress and improved empathy and teamwork	Taylor et al.^23^
Ethics consultation services	Health-system ethics programs	Moral distress consultation services	Reframes distress as a system-level issue and identifies modifiable drivers	Hamric & Epstein^20^

Abbreviation: ICU, intensive care unit.

Moral distress consultation services further operationalize organizational accountability by reframing distress as a signal of system misalignment rather than individual failure. Evaluations show that such services commonly surface chronic contributors, including understaffing, policy ambiguity, and cultural barriers, and support collaborative, system-level problem solving.^20^ In burn units, proactive ethics consultation (eg, scheduled case conferences for high-risk scenarios) may help prevent moral residue and identify modifiable organizational drivers.

Interventions focused primarily on individual resilience have important limitations. Mindfulness, stress-management, and self-care programs may reduce burnout and compassion fatigue in the short term, but they do not address structural drivers such as chronic understaffing, documentation burden, or conflicting performance incentives. A narrow emphasis on resilience risks individualizing what are fundamentally system-level value conflicts and may obscure organizational accountability, particularly in burn care where moral stakes are high and decisions often irreversible.

From an organizational ethics perspective, burn centers and health systems bear obligations in at least 3 domains. First, they must design care systems, including staffing models, triage policies, and resource allocation processes, that minimize foreseeable moral stressors and distribute burdens fairly. Second, they must cultivate ethical climates in which staff at all levels can voice concerns, participate in deliberation, and influence decisions without fear of retaliation. Third, they must provide accessible, well-resourced ethics and peer support services that enable early intervention when moral distress arises. These obligations echo broader ethical frameworks that view the organization itself as a moral agent with responsibilities to both patients and staff.^12^^,^^31^

For burn care specifically, there is a need to embed these principles into accreditation standards, quality metrics, and professional guidelines. Joint Commission requirements for ethics mechanisms already provide a regulatory foothold, but burn societies and trauma organizations could go further by explicitly recognizing moral injury prevention as a component of quality care. Burn centers might, for example, track moral distress or compassion fatigue as part of workforce dashboards, integrate moral injury risk into hazard analyses, and include ethics consultation metrics in quality improvement portfolios. Considering that moral distress contributes to turnover, and turnover threatens the capacity of burn centers to provide high-quality care, investments in organizational ethics infrastructure can be justified not only on moral grounds but also on strategic and economic ones.

### Limitations of the evidence base

This review has several limitations rooted in the underlying literature. Burn-specific empirical data on moral distress, compassion fatigue, and moral injury remain limited in number and sample size. Much of the evidence derives from single-site, cross-sectional surveys with potential response bias, limiting causal inference. Measures of moral distress and moral injury are still evolving, and many studies conflate or overlap constructs such as burnout, secondary traumatic stress, and moral distress.^34^^,^^35^ Intervention studies are often small, short-term, and heterogeneous, making it difficult to compare effectiveness across programs. Finally, relatively few studies directly evaluate the impact of ethics consultation or organizational reforms on patient outcomes or staff retention in burn care. These gaps underscore the need for more rigorous, burn-specific research, including longitudinal studies and controlled evaluations of multilevel interventions.

## CONCLUSION

Moral distress, moral injury, and compassion fatigue among burn care staff are not idiosyncratic failings of individual resilience; they are predictable consequences of practicing in morally charged, resource-constrained systems. Evidence from burn units and analogous high-acuity settings shows that recurrent antecedents, including resource scarcity, triage constraints, perceived futility, role conflict, and unclear authority lines, are organizationally mediated and linked to emotional exhaustion, diminished empathy, turnover intention, and risks to safety and quality. Multilevel organizational measures, including supportive leadership, structured peer support and debriefings, psychologically safe communication, clear escalation pathways, and timely ethics consultation, offer the most robust protection against moral harm. Resilience training alone is insufficient when it is not accompanied by reforms in workload, staffing, and policies. For burn centers, moral injury and compassion fatigue must therefore be understood as organizational ethics problems. Meaningful solutions require institutional accountability and system-level design that reduces preventable distress, strengthens clinicians’ moral agency, and normalizes early ethics input as an integral part of high-quality burn care.

## Notes

Author contributions: Joshua Khorsandi (Writing—original draft [lead]), Jason Mirharooni (Writing—review & editing [equal]), Joshua Ahdout (Methodology [equal]), Justin Kahen (Writing—review & editing [lead]), Eli Nazarian (Formal analysis [equal]), Demitri Franzoni (Supervision [lead]), and Joshua MacDavid (Supervision [equal])

Funding: This research received no external funding.
